# Sumoylation of thymine DNA glycosylase impairs productive binding to substrate sites in DNA

**DOI:** 10.1016/j.jbc.2024.107902

**Published:** 2024-10-18

**Authors:** Lakshmi S. Pidugu, Hardler W. Servius, Kurt B. Espinosa, Mary E. Cook, Kristen M. Varney, Alexander C. Drohat

**Affiliations:** 1 Department of Biochemistry and Molecular Biology, University of Maryland School of Medicine, Baltimore, Maryland, USA; 2 Molecular and Structural Biology Program, University of Maryland Marlene and Stewart Greenebaum Comprehensive Cancer Center, Baltimore, Maryland, USA

**Keywords:** DNA damage, mutation, GT mismatch, base excision repair, enzyme, small ubiquitin-like modifier, sumo-interacting motif, active DNA demethylation, cancer

## Abstract

The base excision repair enzyme thymine DNA glycosylase (TDG) protects against mutations by removing thymine or uracil from guanine mispairs and functions in active DNA demethylation by excising 5-formylcytosine (fC) and 5-carboxylcytosine (caC). Post-translational modification of TDG by SUMO (small ubiquitin-like modifier) reduces its glycosylase activity but the mechanism remains unclear. We investigated this problem using biochemical and biophysical approaches and a TDG construct comprising residues 82 to 340 (of 410) that includes the SUMOylation site and the motif for non-covalent SUMO binding. Single turnover kinetics experiments were collected at multiple enzyme concentrations ([E]) and the hyperbolic dependence of activity (*k*_obs_) on [E] yielded the maximal glycosylase activity (*k*_max_), the enzyme concentration giving half-maximal activity (*K*_0.5_), and the catalytic efficiency (*k*_max_/*K*_0.5_). Sumoylation of TDG (or TDG^82-340^) causes large reductions in catalytic efficiency for G·T, G·U, G·fC, and G·caC DNA substrates, due largely to weakened substrate affinity (increased *K*_0.5_). ^19^F NMR experiments show that sumoylation of TDG^82-340^ reduces productive binding to G·U mispairs and dramatically impairs binding to G·T mispairs. A mutation in the TDG SUMO-interacting motif (SIM), E310Q, shown previously to perturb the noncovalent binding of SUMO to unmodified TDG, rescues the glycosylase activity of sumoylated TDG^82-340^. Similarly, NMR studies show the mutation restores the productive binding of sumoylated TDG^82-340^ to G·U and G·T pairs. Together, the results indicate that intramolecular SUMO-SIM interactions mediate the adverse effect of sumoylation on TDG activity and suggest a model whereby the disruption of SUMO-SIM interactions enables productive binding of sumoylated TDG to substrate sites in DNA.

Post-translational modifications (PTMs) alter the structure and function of proteins and feature a broad range of modifying compounds, ranging from small molecules (*e.g.*, phosphate) to protein domains such as ubiquitin (Ub) or SUMO (small ubiquitin-like modifier). While Ub modification typically signals protein degradation, SUMO conjugation often serves to enhance protein-protein interactions, whereby the SUMO domain conjugated to one protein binds non-covalently to the SUMO-interacting motif (SIM) of another (1). SUMO modification (sumoylation) can also promote signaling events, modulate the effects of other PTMs (*e.g.*, stimulate Ub modification), and mediate the formation of biomolecular condensates through liquid-liquid phase separation (1, 2).

Sumoylation also regulates protein function or enzyme activity (3, 4). A paradigm example is thymine DNA glycosylase (TDG), which initiates base excision repair (BER) by removing damaged or purposefully modified nucleobases from DNA (5, 6). TDG excises base lesions including thymine from G·T mispairs generated *via* deamination of 5-methylcytosine (mC), uracil from G·U mispairs, and 7,8-dihydro-8-oxoadenine (oxoA), among others (7, 8, 9, 10, 11). TDG also removes 5-formylcytosine (fC) and 5-carboxylcytosine (caC), oxidized forms of mC that are generated by TET (ten 11 translocation) enzymes in active DNA demethylation (12, 13, 14). TDG excises bases by catalyzing hydrolysis of the *N*-glycosyl bond, producing an apurinic/apyrimidinic (AP) site, and follow-on BER enzymes complete the repair process. Initial studies reported that SUMO-modified TDG does not bind detectably to DNA containing a substrate site (G·T, G·U) or an abasic product site, suggesting that sumoylation abolishes TDG binding to specific sites in DNA (6, 15, 16). Subsequent work revealed that sumoylation of TDG weakens but does not abolish its binding to DNA substrates (G·T, G·fC, G·caC) and abasic DNA (17, 18). The studies also showed that sumoylation of TDG reduces its glycosylase activity for some substrates, particularly G·T mispairs. However, the mechanism by which sumoylation impairs substrate binding and base excision remained unclear.

Human TDG contains 410 residues and includes a conserved catalytic domain (residues 111–300) flanked by large N- and C-terminal regions that are intrinsically disordered (Fig. 1*A*) (19, 20). The disordered regions mediate interactions with other proteins and contain sites for a variety of PTMs including acetylation, phosphorylation, ubiquitination, and sumoylation (6, 17, 19, 21, 22, 23, 24, 25, 26, 27). SUMO modification of TDG occurs exclusively at one Lys residue (K330) located in a consensus site of the disordered C-terminal region (28). SUMO domains that are conjugated to TDG can bind the SUMO-interacting motif (SIM) of that same TDG molecule through noncovalent SUMO·SIM interactions, as shown by crystal structures of TDG modified by SUMO-1 (Fig. 1, *B* and *C*) or SUMO-3 (15, 16). In its unmodified form, TDG binds non-covalently to free SUMO proteins and to the SUMO domain of other sumoylated proteins including promyelocytic leukemia protein (PML), p53 (and p73), and the histone acetyltransferases CBP/p300, among others (6, 15, 16, 22, 29, 30). Notably, the sumoylation of TDG suppresses its binding to free SUMO proteins, likely because the intramolecular SUMO-SIM interaction must be disrupted (22, 29).

**Figure 1:**
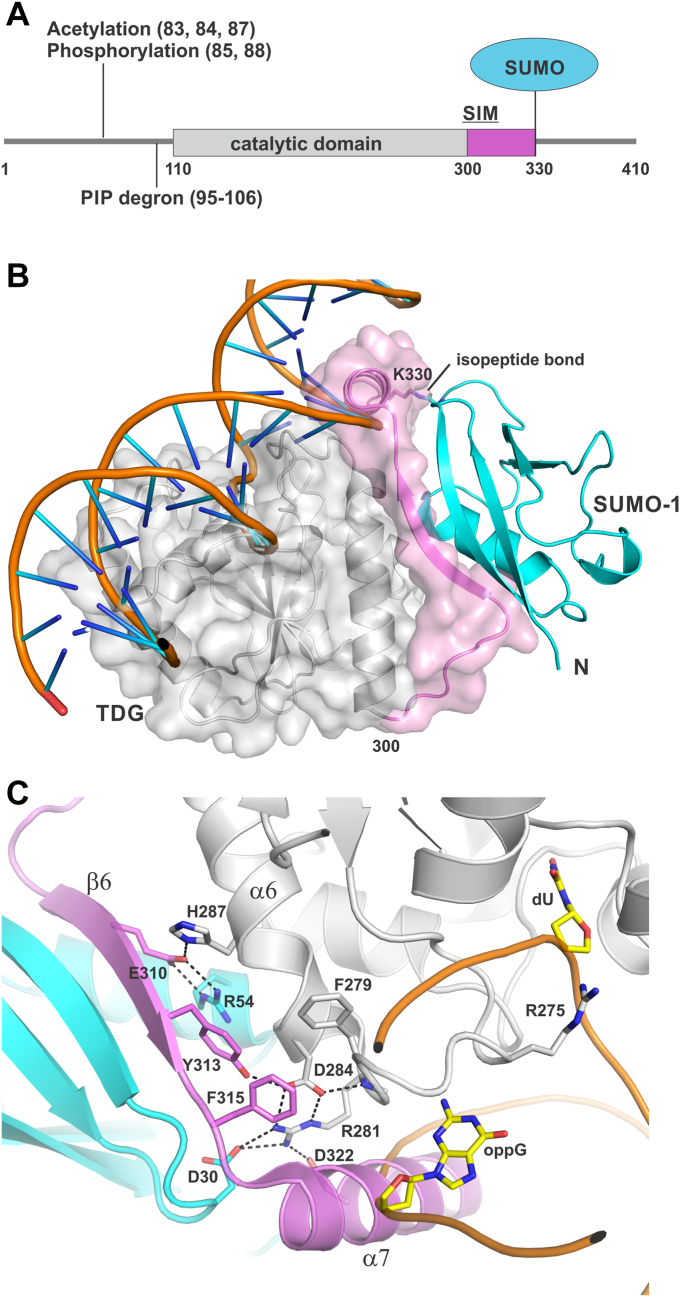
**Primary structure of TDG and crystal structure of TDG conjugated to SUMO-1.***A*, primary structure of TDG denoting the catalytic domain, the sites of some PTMs, and the region containing the SUMO-interacting motif (SIM). *B*, structure of human TDG (residues 117–332) modified by SUMO-1 with coloring as indicated in panel A (PDB ID: 1wyw). Because no structure is available for sumoylated TDG bound to DNA, the DNA was positioned by aligning a structure of DNA-bound TDG (PDB ID: 5HF7, hidden) with that of sumoylated TDG (shown). This model illustrates that helix α7, as positioned in the crystallographic conformation of sumoylated TDG, would pose a steric block to DNA binding, based on the binding mechanism observed in all extant structures of unmodified TDG. *C*, close-up view of the SUMO-SIM interactions in the same structure (different orientation). The SUMO-SIM interactions involve a strand (β6) and residues 279 to 281 and 284 of TDG. Strand β6 contains a canonical SIM comprising residues ^308^VQEV^311^. Some SUMO-SIM interactions are not shown, including backbone hydrogen bonds joining β6 of TDG and the beta-sheet of SUMO-1. For additional perspective, DNA containing a G·U mispair is also shown, with the dU nucleotide (*yellow*) flipped into the active site.

The structures of sumoylated TDG show that intramolecular association of the tethered SUMO domain involves residues in a strand (β6; 307–314), which contains a canonical SIM (V^308^Q^309^E^310^V^311^), as well as residues F279, P280, R281, and D284 of TDG (Fig. 1*C*). Mutation of SUMO-interacting residues weakens the binding of unmodified TDG to free SUMO proteins and to SUMO-modified proteins, most prominently for the R281A, E310Q, and F315A mutations (15, 16, 22). Notably, these mutations were shown to reverse, at least partially, the adverse effect of sumoylation on TDG binding to abasic DNA. The structures of sumoylated TDG reveal a helix in the C-terminal region of TDG (α7; residues 317–329) that protrudes away from its catalytic domain (Fig. 1, *B* and *C*). Although there are no reported structures of sumoylated TDG bound to DNA, modeling suggests helix α7 could perturb the binding of sumoylated TDG to DNA, absent a conformational change (Fig. 1, *B* and *C*) (15, 16). While there are no reported structures of unmodified TDG that include C-terminal residues beyond ∼305, AlphaFold predicts the C-terminal region is disordered except for α7 (residues 317–329) (31, 32). Together, these observations suggest that intramolecular SUMO-SIM interactions mediate the adverse effect of sumoylation on DNA binding and base excision, by stabilizing β6 and α7 in a manner that weakens DNA binding. However, the underlying mechanism remains unclear.

Like most DNA glycosylases, TDG gains access to modified bases in DNA through nucleotide flipping, a large and reversible conformational change that precedes the base excision (chemical) step of the reaction (33) (Fig. 1*C*). Thus, base flipping is required to form a productive enzyme-substrate complex and sumoylation could potentially reduce TDG activity by perturbing base flipping. We previously reported an approach to monitor enzyme-mediated base flipping (*K*_flip_) using fluorine (^19^F) NMR spectroscopy and DNA containing a 2′-flouroarabino-substituted deoxynucleotide, 2′-F-dT (T^F^) or 2′-F-dU (U^F^) (34). ^19^F NMR is a powerful approach for studying conformational change in biological systems given the absence of fluorine in most biomolecules, the 100% natural abundance of ^19^F, and the hypersensitivity of fluorine to its local environment (35). We showed that 1D NMR spectra report on TDG-mediated flipping of T^F^ from a G·T^F^ pair or U^F^ from a G·U^F^ pair in DNA. In the studies reported here, we used ^19^F NMR, single turnover enzyme kinetics experiments, and other biochemical methods to investigate the mechanism by which sumoylation modulates the glycosylase activity of TDG.

## Results

### Approach for determining the catalytic efficiency of TDG

The TDG reaction exhibits very slow product release and strong product inhibition such that the rate constant obtained from multiple turnover kinetics experiments (*k*_cat_), collected with excess substrate relative to the enzyme, is dominated by these events and is not useful for comparing glycosylase activity among different TDG constructs or substrates (36, 37, 38). By contrast, single turnover kinetics experiments, collected with a molar excess of enzyme relative to the substrate, give a rate constant (*k*_obs_) that is not influenced by events after base excision. To determine the effect of sumoylation on the catalytic efficiency of TDG, we sought to collect the experiments with varying enzyme concentration ([E]), including concentrations low enough to reduce *k*_obs_ while maintaining excess enzyme conditions. Under such conditions, fitting the hyperbolic dependence of *k*_obs_ on [E] yields *k*_max_, the maximal rate of glycosylase activity, and *K*_0.5_, the enzyme concentration that gives half-maximal activity. Importantly, the ratio of these parameters, *k*_max_/*K*_0.5_, gives the catalytic efficiency, analogous to *k*_cat_/*K*_M_ obtained from multiple turnover kinetics experiments. However, no previous studies have employed such an approach to determine the catalytic efficiency of TDG (to our knowledge).

**Figure 2:**
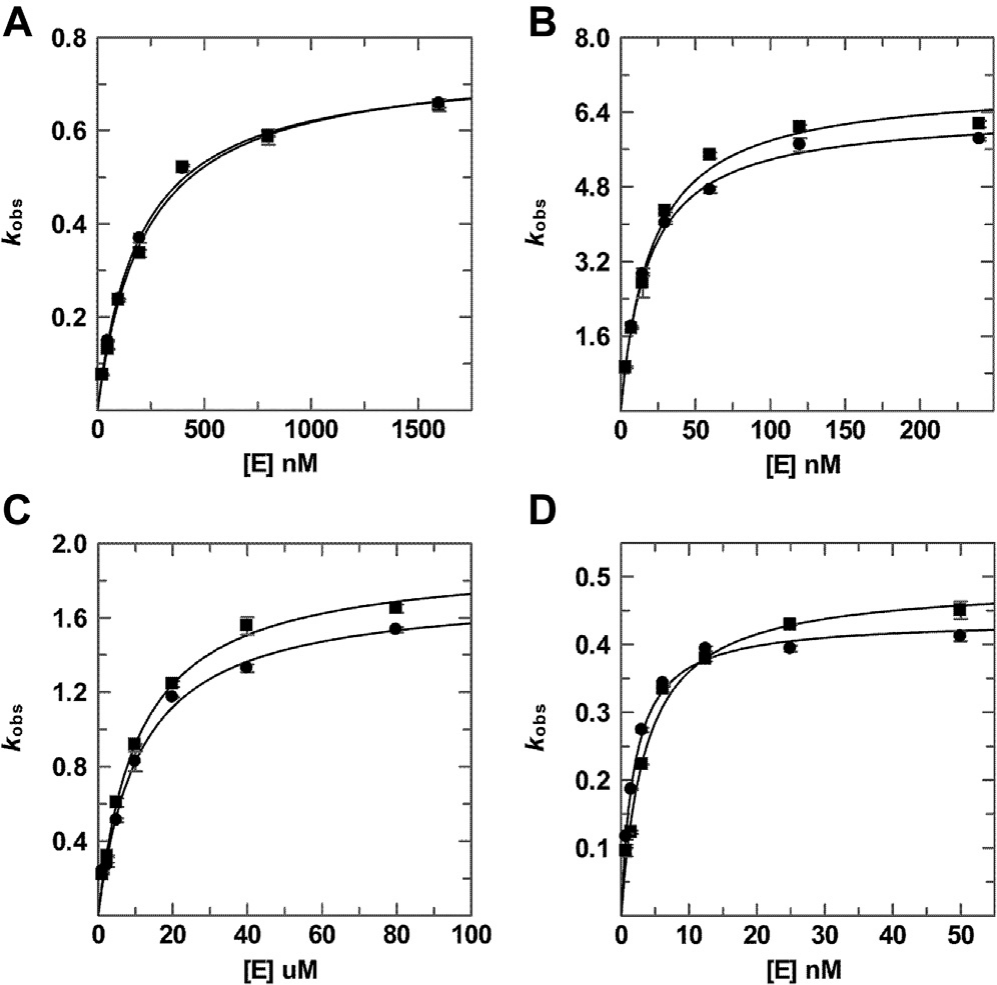
**Glycosylase activity of unmodified TDG and TDG**^**82-340**^**.** Dependence of glycosylase activity (*k*_obs_) on enzyme concentration ([E]) for TDG (*squares*) and TDG^82-340^ (*circles*) acting on DNA substrates including (*A*) G·T, (*B*) G·U, (*C*) G·fC, and (*D*) G·caC. For each of several enzyme concentrations, single turnover experiments were collected (at 37 °C) with a substrate concentration of 0.2 nM and the resulting progress curves were fitted to obtain *k*_obs_ (Figs. S1 and S2). The dependence of *k*_obs_ on [E] was fitted (Equation 2) to obtain the maximal rate constant (*k*_max_) and *K*_0__.5_ (shown in Table 1).

Previous findings that TDG binds tightly to DNA substrates, with a *K*_*d*_ as low as 1 nM (18, 20, 39, 40), indicated that in order to determine the catalytic efficiency (*k*_max_/*K*_0.5_), the single turnover kinetics experiments would need to be collected with low or sub nanomolar DNA concentrations in order to maintain excess enzyme conditions. However, our standard HPLC approach, whereby the DNA (substrate and product) is detected by absorbance (*A*_260_) (41), is not sufficiently sensitive to monitor reactions with less than ∼0.25 μM DNA substrate (in 50 μl sample volume). As such, we used a fluorescence detector for the HPLC analysis and DNA with 3′-fluorescein on the target strand, enabling us to monitor reactions with sub-nanomolar substrate concentrations. The kinetics experiments were performed (at 37 °C) with varying concentrations of TDG and a limiting concentration of 28 bp DNA that contained a G·T, G·U, G·fC, or G·caC base pair. Initial experiments collected using our standard reaction buffer (0.02 M HEPES pH 7, 0.1 M NaCl) indicated that activity (*k*_obs_) did not decrease sufficiently with [TDG] to accurately fit *K*_0.5_ for some substrates (G·fC, G·caC). To address this problem, and more closely reflect physiological conditions, the reactions were collected with 0.15 M NaCl and 250 nM nonspecific DNA (28 bp). Under these conditions, fitting the hyperbolic dependence of *k*_obs_ on [TDG] for a G·T substrate gives *k*_max_ = 0.75 ± 0.02 min^−1^, *K*_0.5_ = 0.22 ± 0.02 μM, and *k*_max_/*K*_0.5_ = 3.4 ± 0.4 μM^−1^·min^−1^ (Figs. 2 and S1 and Table 1). By comparison, maximal activity is nearly tenfold higher for a G·U substrate, *k*_max_ = 6.99 ± 0.23 min^−1^, consistent with prior reports (7, 42), while the catalytic efficiency is 99-fold higher, *k*_max_/*K*_0.5_ = 335 ± 41 μM^−1^·min^−1^. The G·fC substrate exhibits maximal activity of *k*_max_ = 1.91 ± 0.04 min^−1^ and catalytic efficiency of *k*_max_/*K*_0.5_ = 177 ± 13 μM^−1^·min^−1^. While the G·caC substrate exhibits the lowest maximal activity, *k*_max_ = 0.49 ± 0.01 min^−1^, it still has high efficiency, *k*_max_/*K*_0.5_ = 135 ± 15 μM^−1^·min^−1^ since it exhibits the lowest *K*_0.5_. Our results reveal that differences among the four substrates can be much larger for *k*_max_/*K*_0.5_ than for *k*_max_. For example, compared to the results for G·T, *k*_max_ is 1.5-fold lower for G·caC while *k*_max_/*K*_0.5_ is 40-fold higher, due to the large (62-fold) difference in *K*_0.5_ for these substrates.

**Table 1:** Glycosylase activity

Enzyme	Substrate	*k*_max_ (min^−1^)	*K*_0.5_ (μM)	*k*_max_/*K*_0.5_ (μM^−1^·min^−1^)	Relative to *k*_max_ for unmodified TDG	Relative to *k*_max_/*K*_0.5_ for unmodified TDG
TDG	G·T	0.75 ± 0.02	0.222 ± 0.022	3.39 ± 0.35		
	G·U	6.99 ± 0.23	0.021 ± 0.002	335 ± 41		
	G·fC	1.91 ± 0.04	0.011 ± 0.001	177 ± 13		
	G·caC	0.49 ± 0.01	0.0036 ± 0.0004	135 ± 15		
TDG^82-340^	G·T	0.75 ± 0.01	0.201 ± 0.012	3.70 ± 0.23		
	G·U	6.38 ± 0.13	0.019 ± 0.002	343 ± 27		
	G·fC	1.75 ± 0.06	0.011 ± 0.001	156 ± 17		
	G·caC	0.44 ± 0.01	0.0019 ± 0.0002	224 ± 20		
S1∼TDG	G·T	0.0147 ± 0.0004	9.9 ± 0.7	0.0015 ± 0.0001	1/51	1/2279
	G·U	4.14 ± 0.12	6.6 ± 0.4	0.63 ± 0.04	1/1.7	1/535
	G·fC	0.69 ± 0.02	3.4 ± 0.3	0.21 ± 0.02	1/2.8	1/855
	G·caC	0.37 ± 0.02	0.76 ± 0.11	0.48 ± 0.07	1/1.3	1/279
S1∼TDG^82-340^	G·T	0.047 ± 0.006	14.7 ± 4.0	0.0032 ± 0.0010	1/16	1/1160
	G·U	5.67 ± 0.19	9.2 ± 0.8	0.62 ± 0.06	1/1.1	1/555
	G·fC	1.23 ± 0.03	7.2 ± 0.5	0.17 ± 0.01	1/1.4	1/920
	G·caC	0.43 ± 0.03	2.2 ± 0.4	0.19 ± 0.04	1.0	1/1167
E310Q-TDG^82-340^	G·T	0.75 ± 0.02	0.16 ± 0.02	4.7 ± 0.5		
	G·U	6.64 ± 0.14	0.0070 ± 0.0007	943 ± 90		
S1∼E310Q-TDG^82-340^	G·T	0.69 ± 0.03	0.57 ± 0.08	1.20 ± 0.17	1/1.1	1/3.9
	G·U	5.88 ± 0.07	0.068 ± 0.003	86.7 ± 3.7	1/1.1	1/11

### New TDG construct for studying sumoylation

Because native TDG contains large N- and C-terminal disordered regions (∼110 residues each) we sought a smaller construct that would be more amenable to ^19^F NMR while still including the regions needed for full catalytic activity, the SUMO conjugation site (K330), and the SIM. Our prior studies show that TDG^82-308^ exhibits substrate binding and glycosylase activity equivalent to TDG for DNA containing G·T and G·fC pairs (20, 43). We predicted that a larger construct, TDG^82-340^, which includes the SIM and sumoylation site, could recapitulate the catalytic activity of TDG and the effects of sumoylation on its activity. We note that the construct used for solving crystal structures of sumoylated TDG (15, 16) included residues 111 to 339 but lacked N-terminal residues (82–110) that are important for substrate binding and glycosylase activity (20, 43). We expressed TDG^82-340^ in *E coli*, purified it to homogeneity, and characterized its glycosylase activity for the same four substrates (G·T, G·U, G·fC, G·caC). We find that the maximal activity (*k*_max_) and catalytic efficiency (*k*_max_/*K*_0.5_) of TDG^82-340^ are highly similar to that of TDG for all four substrates (Figs. 2 and S2 and Table 1), indicating that TDG^82-340^ provides a suitable construct for studying the effects of sumoylation on TDG activity.

### Effect of sumoylation on the activity of TDG

**Figure 3:**
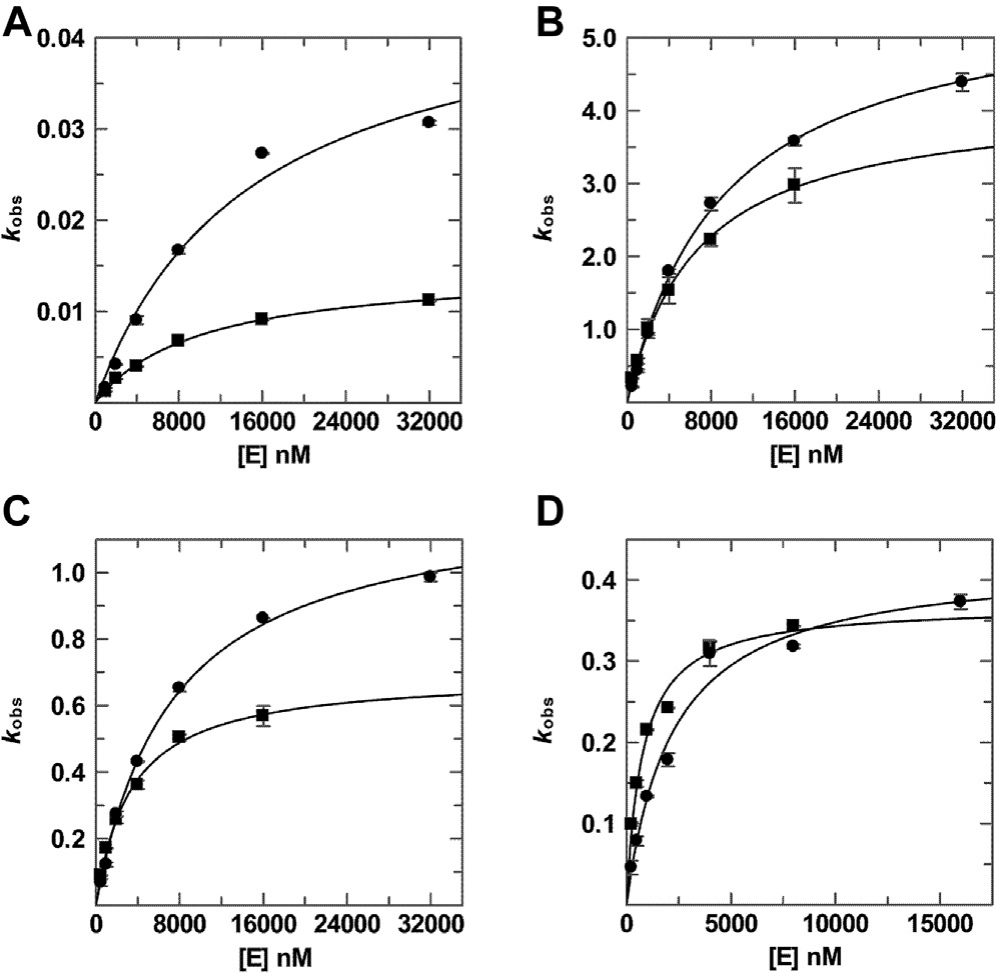
**Glycosylase activity of sumoylated TDG and TDG^82-340^.** Dependence of glycosylase activity (*k*_obs_) on enzyme concentration ([E]) for sumoylated TDG (*squares*) and sumoylated TDG^82-340^ (*circles*) acting on DNA substrates including (*A*) G·T, (*B*) G·U, (*C*) G·fC, and (*D*) G·caC. For each of several enzyme concentrations, single turnover experiments were collected (at 37 °C) with a substrate concentration of 1.6 nM, and the resulting progress curves were fitted to obtain *k*_obs_ (Figs. S3 and S4). The dependence of *k*_obs_ on [E] was fitted (Equation 2) to obtain the maximal rate constant (*k*_max_) and *K*_0.5_ (shown in Table 1).

TDG can be modified by three SUMO isoforms, including SUMO-1, SUMO-2, and SUMO-3 (6, 15, 16). The latter two are nearly identical in amino acid sequence and share about 48% sequence identity with SUMO-1 (44). Previous studies indicate that the effects of sumoylation on TDG structure and activity are similar for modification by SUMO-1 or SUMO-2/3 (15, 16, 18). As such, the studies here focus on TDG and TDG^82-340^ modified by SUMO-1 (hereafter S1∼TDG or S1∼TDG^82-340^). We generated modified TDG in bacteria (*Escherichia coli*) by co-transforming a plasmid for TDG (or TDG^82-340^) and a plasmid for expressing human SUMO-1 (mature form) and the SUMO-activating (E1) and SUMO-conjugating (E2) enzymes, and purified S1∼TDG (and S1∼TDG^82-340^) essentially as described (17, 45). We performed single turnover kinetics experiments, with varying S1∼TDG concentrations, for each of the four DNA substrates (Figs. 3 and S3 and Table 1). We find that sumoylation imparts a large effect on the maximal activity of TDG for G·T substrates, reducing *k*_max_ by 51-fold, and an even larger effect on catalytic efficiency, reducing *k*_max_/*K*_0.5_ by 2280-fold, due predominantly to weakened substrate affinity (increased *K*_0.5_). For the other three substrates (G·U, G·fC, G·caC), sumoylation has a much smaller effect on maximal activity, with *k*_max_ reductions of 1.3- to 2.8-fold, but still exerts a very large effect on catalytic efficiency, with reductions in *k*_max_/*K*_0.5_ of 279- to 855-fold. Again, the loss in catalytic efficiency reflects very large reductions in substrate affinity. S1∼TDG exhibits the highest activity (*k*_max_ and *k*_max_/*K*_0.5_) for the G·U substrate, as observed for unmodified TDG, and the lowest activity for the G·T substrate. Notably, sumoylation enhances the disparity in activity for these two substrates, where *k*_max_ and *k*_max_/*K*_0.5_ are 282- and 420-fold greater, respectively, for G·U relative to G·T. We performed the same series of experiments for S1∼TDG^82-340^ and find that sumoylation reduces *k*_max_ by 16-fold for G·T pairs while it has very small (≤1.4-fold) effects on *k*_max_ for the other substrates (Figs. 3 and S4 and Table 1). However, the effects of sumoylation on the catalytic efficiency of TDG^82-340^ are very large for all four substrates, with reductions in *k*_max_/*K*_0.5_ of 555- to 1167-fold, owing to large reductions in substrate affinity. Sumoylated TDG^82-340^ exhibits the highest activity (*k*_max_ and *k*_max_/*K*_0.5_) for the G·U substrate, as observed for unmodified TDG^82-340^, and the lowest activity for the G·T substrate. Together, the results reveal that sumoylation greatly impairs the ability of TDG to bind productively to substrate sites in DNA, and that sumoylation exerts similar effects on the activity of TDG and TDG^82-340^.

### ^19^F NMR studies of base flipping by TDG^82-340^

**Figure 4:**
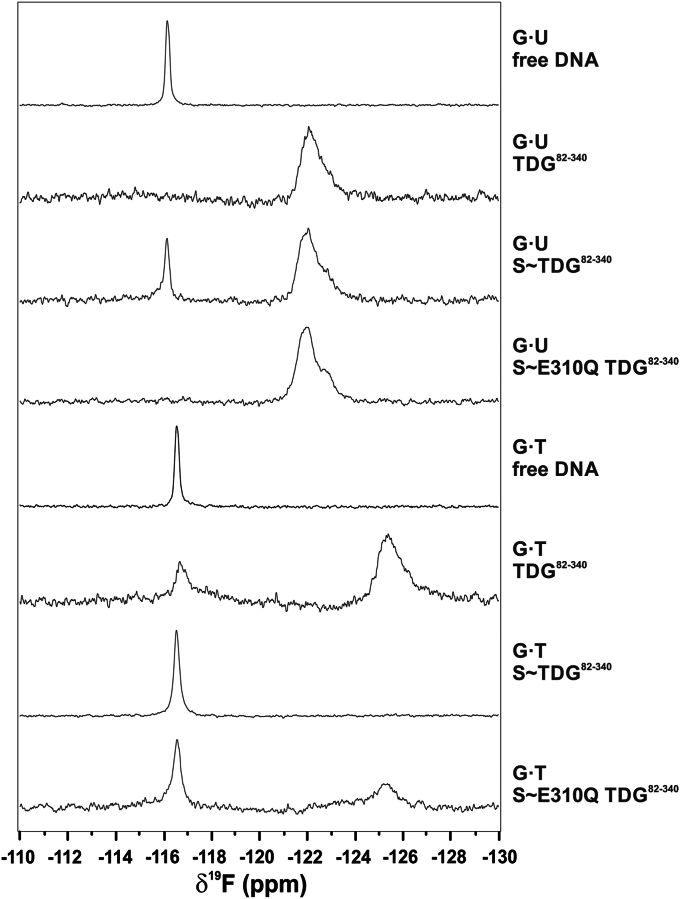
^**19**^**F NMR studies of nucleotide flipping for unmodified and sumoylated forms of TDG**^**82-340**^. Shown are ^19^F NMR spectra for DNA containing a G·U^F^ or a G·T^F^ base pair, in the absence or presence of a specified TDG construct, collected at 30 °C. Labels (*right*) indicate the type of DNA and enzyme (if present) in the sample. The DNA concentration was 45 μM for free G·U^F^ DNA and 50 μM for free G·T^F^ DNA. The remaining samples contained a DNA concentration of 58 to 75 μM and a 2.7- to 3.0-fold higher concentration of the specified TDG construct.

We next sought to investigate the mechanism by which sumoylation reduces the glycosylase activity of TDG. We previously showed that nucleotide flipping can be directly monitored using ^19^F NMR and DNA containing a 2′-flouroarabino deoxynucleotide, including 2′-F-dT (T^F^) or 2′-F-dU (U^F^) (34). In addition, 2′-F-substituted deoxynucleotides are powerful tools for structural and biochemical studies of TDG and other DNA glycosylases, because the subtle 2′-F substitution precludes hydrolysis of the *N*-glycosyl bond (39, 46, 47, 48). Thus, the 2′-F substitution enables studies of a stable enzyme-substrate complex, where the nucleotide flips into the active site but is not cleaved (20, 43, 49, 50, 51). Importantly, our prior studies show that the 2′-F-substitution does not substantially alter the conformation of the flipped nucleotide or its interactions with the TDG active site (for 2′-F-5-carboxyl-dC) (52). In the current studies, we used ^19^F NMR to determine the effect of sumoylation on nucleotide flipping by TDG^82-340^ for DNA containing a G·U^F^ or G·T^F^ mispair (Fig. 4). The spectra were collected at 30 °C (rather than 37 °C) to optimize the stability of the enzyme during the long NMR experiments (up to 10 h). The spectrum for free G·U^F^ DNA shows a single peak (δ^19^F −116.2 ppm), consistent with findings that the nucleotides in a G·U wobble base pair are predominantly stacked in the DNA duplex, even as they experience rapid opening and closing kinetics (34, 53, 54, 55). The spectrum of G·U^F^ DNA bound to TDG^82-340^ exhibits a single peak (δ^19^F −122.1 ppm) that is much broader and shifted upfield by 5.9 ppm relative to the peak for free DNA. These observations indicate that the G·U^F^ DNA is fully bound to TDG^82-340^ and that the mismatched U^F^ is flipped into the active site with no detectable population of U^F^ that remains stacked in the duplex. This finding is fully consistent with our prior observations for G·U^F^ DNA bound to either of two smaller TDG constructs, TDG^82-308^ and TDG^111-308^ (single peak, shifted 6 ppm upfield relative to free G·U^F^ DNA) (34). We note that the assignment of peaks to the stacked or flipped state for G·U^F^ and G·T^F^ DNA was described previously (34).

We performed the same experiments for DNA with a G·T^F^ mismatch and the spectrum for free G·T^F^ DNA shows a single peak (δ^19^F −116.6 ppm), indicating that T^F^ is stacked in the DNA duplex, consistent with expectations (as noted above for G·U^F^ DNA) (Fig. 4). The spectrum for G·T^F^ DNA bound to a threefold molar excess of TDG^82-340^ exhibits two broadened peaks, one of which has nearly the same chemical shift (δ^19^F −116.7 ppm) observed for free G·T^F^ DNA and corresponds to T^F^ in the stacked state for enzyme-bound DNA. The other peak (δ^19^F −125.4 ppm), positioned 8.7 ppm upfield, reflects T^F^ flipped into the TDG active site. Given that the concentration of TDG^82-340^ in the NMR sample (225 μM) is 1125-fold higher than the *K*_0.5_ for TDG^82-340^ acting on G·T DNA (0.20 μM; Table 1), the population of free G·T^F^ DNA in the NMR sample is expected to be very low. Notably, previous ^19^F NMR studies of G·T^F^ DNA bound to a smaller construct (TDG^82-308^) also showed two peaks separated by 8.7 ppm, and the peak for stacked T^F^ was similarly broadened for TDG-bound *versus* free G·T^F^ DNA (34). Moreover, broadening of the peak for stacked T^F^ was observed for TDG-bound DNA with an A·T^F^ pair, for which no T^F^ flipping is expected. As we suggested previously, peak broadening for stacked T^F^ can be explained by the increased rotational correlation time for the 28 bp DNA (17 kDa) upon binding to TDG^82-340^ (31 kDa), which increases the ^19^F transverse relaxation rate (*R*_2_). Broadening of the peak for stacked T^F^ could also potentially arise from exchange of the DNA between free and TDG-bound states, or possibly the exchange involving multiple nonspecific TDG binding sites on the DNA. Considering the data for G·T^F^ DNA bound to TDG^82-340^, the peak integrals for flipped and stacked T^F^ (*I*_F_/*I*_S_ = 2.6) indicate that about 72% of the T^F^ is flipped into the active site and about 28% remains stacked in the duplex.

### Sumoylation impairs productive substrate binding by TDG

To investigate the effect of sumoylation on substrate binding, we collected ^19^F NMR spectra for G·U^F^ DNA with a 3-fold molar excess of S1∼TDG^82-340^. The spectrum has a large peak corresponding to U^F^ that is flipped into the active site (δ^19^F −122.1 ppm) and a smaller peak corresponding to U^F^ that is stacked in the DNA duplex (δ^19^F −116.2 ppm). While the peak integrals (*I*_F_/*I*_S_ = 3.9) indicate that the mismatched U^F^ is predominantly flipped, the presence of a peak for U^F^ in the stacked state indicates that sumoylation substantially reduces the ability of TDG to bind productively to a G·U^F^ pair in DNA, with U^F^ flipped into the active site. Remarkably, the NMR spectrum for G·T^F^ DNA bound to sumoylated TDG^82-340^ exhibits only a single peak, with a chemical shift corresponding to T^F^ in the stacked state (δ^19^F −116.5 ppm). The absence of a detectable peak for T^F^ in the flipped state demonstrates that sumoylation greatly impairs the capacity of TDG to bind productively to a G·T^F^ pair in DNA. Thus, the NMR result is consistent with the very large decreases in substrate affinity (*K*_0.5_) and catalytic efficiency (*k*_max_/*K*_0.5_) caused by sumoylation of TDG^82-340^. It is notable that the peak for stacked T^F^ is substantially broadened when G·T^F^ DNA binds unmodified TDG^82-340^ but not when it binds sumoylated TDG^82-340^, even as sumoylation increases the molecular weight of TDG by 12 kDa. One potential explanation is that the lifetime of the nonspecific TDG-DNA complex (in which T^F^ is stacked) is much shorter for sumoylated relative to unmodified TDG, such that *R*_2_ for G·T^F^ DNA is not dramatically enhanced by transient binding to sumoylated TDG. Alternatively, we cannot exclude the possibility that some fraction of the G·T^F^ DNA is free in the NMR sample with sumoylated TDG^82-340^, which could potentially occur if the protein self-associates or binds DNA with a stoichiometry greater than 2:1.

### Disrupting SUMO-SIM interactions restores glycosylase activity for sumoylated TDG

**Figure 5:**
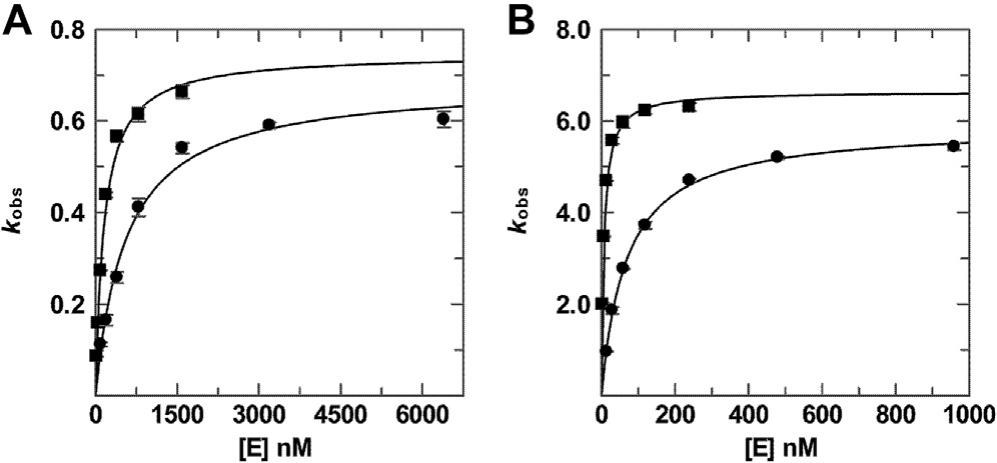
**Glycosylase activity of unmodified and sumoylated E310Q-TDG^82-340^.** Dependence of glycosylase activity (*k*_obs_) on enzyme concentration ([E]) for E310Q-TDG^82-340^ unmodified (*circles*) and SUMO-1-modified (*squares*) for DNA substrates including (*A*) G·T and (*B*) G·U. For each of several enzyme concentrations, single turnover experiments were collected (at 37 °C) with a substrate concentration of 0.2 nM and the resulting progress curves were fitted to obtain *k*_obs_ (Figs. S5 and S6). The dependence of *k*_obs_ on [E] was fitted (Equation 2) to obtain the maximal rate constant (*k*_max_) and *K*_0.5_ (shown in Table 1).

Previous studies found that mutations in the SIM region of TDG, including E310Q, disrupt SUMO-SIM interactions and weaken the binding of free SUMO proteins to unmodified TDG (15, 16). Moreover, the mutations were found to partially reverse the adverse effect of sumoylation on TDG binding to abasic DNA. However, no prior studies have reported whether SIM mutations reverse the loss in glycosylase activity caused by sumoylation. To address this problem, we produced TDG^82-340^ with the E310Q mutation. We selected this particular mutation because E310 resides in the four-residue SIM of TDG (V^308^Q^309^E^310^V^311^) and it forms a bidentate salt bridge with a conserved Arg residue of SUMO-1 and SUMO-2/3 (Fig. 1*C*) (15, 16). We anticipated that the E310Q mutation would not have a large effect on the activity of on unmodified TDG^82-340^, given that E310 is distal from the active site. To test this idea, we determined the concentration dependence of E310Q-TDG^82-340^ activity for G·T and G·U substrates, which exhibit the lowest and highest catalytic efficiency among the four substrates studied in this work. We find that the E310Q mutation does not alter the maximal activity (*k*_max_) of unmodified TDG^82-340^ and confers a very minor increase in catalytic efficiency (*k*_max_/*K*_0.5_) of 1.3- and 2.8-fold for G·T and G·U substrates, respectively (Figs. 5 and S5 and Table 1). We then generated sumoylated E310Q-TDG^82-340^ in *E coli*, purified it to homogeneity, and determined its glycosylase activity (Figs. 5 and S6 and Table 1). Remarkably, we find that the E310Q mutation nearly eliminates the effect of sumoylation on TDG activity for a G·T substrate. Indeed, *k*_max_ is essentially unchanged and *k*_max_/*K*_0.5_ is reduced by only 3.9-fold by sumoylation of E310Q-TDG^82-340^, compared to the 16- and 1160-fold reductions in these parameters for sumoylation of wild-type TDG^82-340^. While *k*_max_ for the G·U substrate is not substantially altered by sumoylation of wild-type or E310Q-TDG^82-340^, sumoylation reduces the catalytic efficiency by 563-fold for wild-type TDG^82-340^ but only 11-fold for E310Q TDG^82-340^. Thus, the kinetics results indicate that the activity impairment caused by sumoylation is largely rescued by the E310Q mutation. These observations indicate that intramolecular SUMO-SIM interactions, which are at least partially disrupted by the E310Q mutation, mediate the adverse impact of sumoylation on TDG catalytic activity.

### Disrupting SUMO-SIM interactions restores productive substrate binding for sumoylated TDG

We also used ^19^F NMR to examine how the E310Q mutation modulates the effect of sumoylation on substrate binding by TDG. Remarkably, the spectrum for G·U^F^ DNA bound to S1∼E310Q-TDG^82-340^ exhibits a single peak corresponding to U^F^ flipped into the active site (δ^19^F −122.1 ppm), with no detectible peak for U^F^ stacked in the duplex (Fig. 4). Indeed, the spectrum for sumoylated E310Q-TDG^82-340^ is essentially the same as that of unmodified TDG^82-340^. Thus, the reduction in U^F^ flipping caused by sumoylation of TDG^82-340^ is rescued by the E310Q mutation. The spectrum for G·T^F^ DNA bound to sumoylated E310Q-TDG^82-340^ exhibits two peaks, one for T^F^ that is flipped into the active site (δ^19^F −125.4 ppm) and the other for stacked T^F^ (δ^19^F −116.6 ppm). The ratio of peak integrals (*I*_F_/*I*_S_ = 0.8) is much closer to that of unmodified TDG^82-340^ (*I*_F_/*I*_S_ = 2.6) than to S1∼TDG^82-340^ (*I*_F_/*I*_S_ < 0.03, assuming a peak integral <3% is not detected). Thus, the NMR results indicate that the E310Q mutation largely rescues the loss in binding to G·T mispairs caused by sumoylation of TDG^82-340^.

## Discussion

We investigated the mechanism by which sumoylation reduces the glycosylase activity of TDG using single turnover enzyme kinetics, mutagenesis, and ^19^F NMR spectroscopy. We developed a modified HPLC approach to perform single turnover kinetics experiments using sub-nanomolar DNA substrate concentrations, enabling us to fit the dependence of glycosylase activity (*k*_obs_) on enzyme concentration and thereby determine the maximal activity (*k*_max_) and catalytic efficiency (*k*_max_/*K*_0.5_) for unmodified and sumoylated TDG. The results show that the effect of sumoylation on *k*_max_ is small (<3-fold) for G·U, G·fC, and G·caC substrates but large (51-fold) for G·T substrates. However, sumoylation dramatically impairs the productive binding of TDG to all four substrates, as indicated by massive reductions in catalytic efficiency (*k*_max_/*K*_0.5_) due to large increases in *K*_0.5_. The kinetics results also show that the effects of sumoylation on glycosylase activity are similar for TDG and TDG^82-340^. The ^19^F NMR results indicate that sumoylation reduces the ability of TDG^82-340^ to bind a G·U^F^ pair with U^F^ flipped into the active site and precludes detectable binding to a G·T^F^ pair with T^F^ flipped. While the very weak G·T glycosylase activity observed for sumoylated TDG requires at least transient formation of a productive enzyme-substrate (E·S) complex, with thymine flipped into the active site, the NMR results indicate that formation of this complex is dramatically impaired by sumoylation. Thus, our findings indicate that sumoylation of TDG greatly diminishes its capacity to bind productively to a substrate base pair in DNA, with the largest effect for G·T pairs.

We also investigated the role of intramolecular SUMO-SIM interactions in mediating the effect of sumoylation on TDG activity. Structures of sumoylated TDG show that the tethered SUMO domain binds the SIM of TDG and helix α7 adopts a conformation that would appear to perturb DNA binding (Fig. 1) (15, 16). Previous studies indicate that mutation of TDG residues that contact a tethered SUMO domain (including R281A, E310Q, F315A) weakens the binding of unmodified TDG to free SUMO proteins (15, 16, 22). The mutations were also shown to mitigate the adverse effect of sumoylation on TDG binding to abasic DNA. However, prior studies did not investigate whether or how these mutations might impact the catalytic activity of sumoylated TDG. Our enzyme kinetics experiments reveal that the adverse effects of sumoylation on catalytic efficiency (*k*_max_/*K*_0.5_) are greatly diminished by the E310Q mutation, for the G·U and G·T substrates. Similarly, our ^19^F NMR results show that the mutation restores the ability of sumoylated TDG to bind productively to these substrate sites in DNA, with the target base flipped into its active site. Given that the E310Q mutation weakens the binding of TDG to free SUMO proteins, it seems likely that it will also weaken intramolecular SUMO-SIM binding for sumoylated TDG. As such, a tethered SUMO domain may be more prone to dissociate from the SIM for E310Q-TDG relative to wild-type TDG. Together, these observations indicate that the impaired ability of sumoylated TDG to form a productive E·S complex is mediated by intramolecular SUMO-SIM binding, which presumably stabilizes helix α7 and thereby perturbs DNA binding.

**Figure 6:**
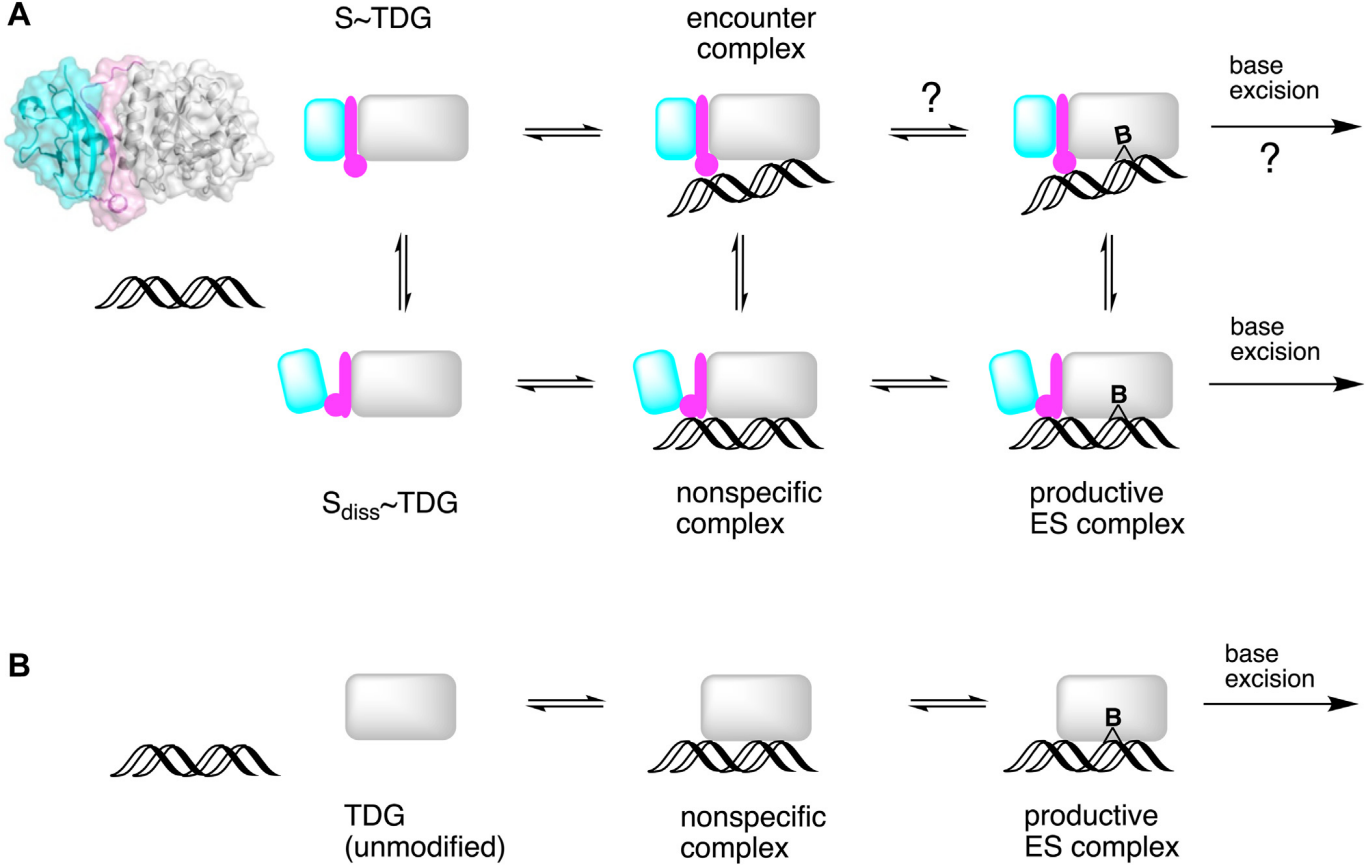
**Modelfor DNA substrate binding by sumoylatedTDG.***A*, the proposed model for substrate binding by sumoylated TDG involves a conformational equilibrium between S∼TDG and S_diss_∼TDG. The S∼TDG conformation reflects the crystal structure of TDG modified by SUMO-1 (*upper left*; PDB ID: 1wyw), where the intramolecular SUMO domain (*cyan*) associates with the SIM of TDG (*magenta*) and helix α7 of TDG (*magenta circle*) perturbs DNA binding. S_diss_∼TDG is a hypothetical conformation in which the tethered SUMO domain dissociates from the SIM and α7 is repositioned (or destabilized) such that it does not perturb DNA binding. S∼TDG likely predominates in the absence of DNA, given the stabilizing effect of intramolecular SUMO-SIM interactions. Our NMR results suggest S∼TDG forms a transient encounter complex with nonspecific DNA (see main text); it seems unlikely that S∼TDG can form a productive E·S complex to enable base excision. The S_diss_∼TDG conformer is expected to be stabilized (relative to free S_diss_∼TDG) by forming a nonspecific DNA complex and stabilized further in a productive E·S complex with a substrate base flipped into its active site. The population of S_diss_∼TDG, free and DNA-bound, is likely enhanced by the E310Q mutation, which weakens the non-covalent binding of SUMO to TDG. It is envisioned that enzyme-DNA interactions in the nonspecific and E·S complexes for S_diss_∼TDG are analogous to those of unmodified TDG (*B*), but the S_diss_∼TDG complexes are less stable because the enzyme can convert to S∼TDG.

The results reported here, together with previous observations, point to a model whereby sumoylated TDG adopts multiple conformations involving the SUMO-SIM interface and helix α7 (Fig. 6*A*). In the crystallographic conformation, termed S∼TDG, the intramolecular SUMO domain binds to the SIM of TDG and positions helix α7 in a manner that interferes with DNA binding and thereby base flipping (Fig. 1, *B* and *C*). In an alternative (hypothetical) conformation, termed S_diss_∼TDG, SUMO-SIM interactions are disrupted, the tethered SUMO domain dissociates from the SIM, and helix α7 is repositioned (or destabilized) such that it does not interfere with DNA binding. The S∼TDG conformer seems likely to predominate in the absence of DNA, given the stabilizing effect of SUMO-SIM interactions. In this proposed model, the binding of S∼TDG to DNA gives an encounter complex that is expected to be weakened by helix α7. Through a conformational change, the encounter complex transitions to a nonspecific complex involving S_diss_∼TDG bound to DNA without interference from helix α7. The nonspecific complex could potentially arise through the direct association of DNA and S_diss_∼TDG, depending on its population. The nonspecific complex can progress to a productive E·S complex with the target base flipped into the active site, enabling base excision. It seems unlikely that the encounter complex will transition to a complex involving S∼TDG with a target base flipped, but this cannot be excluded. It is envisioned that the nonspecific and E·S complexes for S_diss_∼TDG are analogous to those for unmodified TDG, regarding protein-DNA interactions, but are of lower stability because S_diss_∼TDG can convert to S∼TDG (Fig. 6*B*). The observation that the E310Q mutation weakens the binding of unmodified TDG to free SUMO proteins suggests that the mutation could also shift the conformational equilibrium of sumoylated TDG toward the S_diss_∼TDG conformer, for free and DNA-bound enzyme. Our findings that the E310Q mutation restores base flipping, as judged by ^19^F NMR, and restores glycosylase activity (*k*_max_ and *k*_max_/*K*_0.5_) for sumoylated TDG lends support to this idea and to our proposal that a productive E·S complex for sumoylated TDG involves a SUMO-dissociated conformation (S_diss_∼TDG). As noted above, the NMR results indicate that binding to nonspecific DNA is weak for sumoylated TDG, in keeping with structural observations that helix α7 is poised to perturb DNA binding. It seems likely that sumoylated TDG binds to nonspecific DNA predominantly as S∼TDG rather than S_diss_∼TDG, where the intramolecular SUMO-SIM interactions of S∼TDG overcome the enhanced protein-DNA interactions afforded by S_diss_∼TDG. Thus, considering the model in Figure 6*A*, our findings suggest that the DNA-bound form of sumoylated TDG partitions mainly between a transient encounter complex (S∼TDG·DNA) and an E·S complex involving S_diss_∼TDG, with a sparse population of a potential intermediary nonspecific complex (S_diss_∼TDG·DNA).

Our results also indicate that the propensity of sumoylated TDG to form a productive E·S complex depends strongly on the substrate base pair. For G·T mispairs, the productive E·S complex appears to be sparsely populated given that it is not detected by ^19^F NMR. It seems that the formation of the putative S_diss_∼TDG conformer, which would exhibit disrupted SUMO-SIM interactions, is poorly stabilized by flipping of thymine into the active site. Previous studies show that the flipping of thymine is sterically hindered by its own methyl group and that of a conserved Ala in the TDG active site (34, 42, 56, 57). A role for steric hindrance in weakening the productive binding of sumoylated TDG to G·T pairs is supported by our finding that the modified enzyme binds much more productively G·U pairs, and U lacks the C5-methyl of T. Notably, our results also show that the adverse effect of steric hindrance on the binding of sumoylated TDG to G·T mispairs is relieved by disrupting SUMO-SIM interactions (E310Q mutation), which likely stabilizes the putative S_diss_∼TDG conformer. In contrast to G·T pairs, our kinetics results (*k*_cat_/*K*_0.5_) show that the S_diss_∼TDG conformer is much more effectively stabilized in a productive E·S complex with the three other substrates (G·U, G·fC, G·caC), and the NMR results support this conclusion for the G·U substrate. Taken together, our results indicate that the propensity of sumoylated TDG to form a productive E·S complex depends on the extent to which the stabilizing effect of base flipping offsets the destabilizing effect of SUMO-SIM interactions. Additional structural and biophysical studies will be needed to test this model. For example, a structure of sumoylated TDG bound in a productive E·S complex, or bound to abasic DNA product, could inform whether the intramolecular SUMO domain dissociates from the SIM and if so, whether it adopts a stable conformation. It could also be insightful to determine the binding affinity of free SUMO to unmodified TDG and characterize the strength of intramolecular SUMO-SIM interactions for sumoylated TDG.

## Experimental procedures

### Enzymes

The plasmid (pJ401) for expressing TDG^82-340^, a construct of human TDG containing residues Ser^82^-Ala^340^, with an N-terminal poly-His tag, was obtained from ATUM, as was the plasmid for the E310Q variant. Wild-type TDG^82-340^ and the E310Q mutant were expressed in *E. coli* BL21(DE3) (at 22 °C) and purified (at 4 °C) using Ni-affinity, ion-exchange (SP sepharose) and size exclusion chromatography as previously described for TDG^82-308^ (20). Sumoylated forms of TDG^82-340^, E310Q-TDG^82-340^, and full-length TDG were generated in *E. coli* co-transformed with the appropriate TDG expression plasmid and a plasmid for expressing human SUMO-1 (mature form), the activating E1 (SAE1-SAE2) enzyme, and the conjugating E2 (Ubc9) enzyme, as described (17, 45). Sumoylated TDG was purified using a Ni-affinity column, an SP Sepharose column, and a Capto S column. Sumoylated forms of TDG^82-340^ and E310Q-TDG^82-340^ were purified using a Ni-affinity column, an SP Sepharose column, a Capto S column (TDG^82-340^) or Capto Q column (E310Q-TDG^82-340^), and a Superdex 200 SEC column (Cytiva). The N-terminal 6x-His tag was not removed from the sumoylated or unmodified forms of the enzymes. The purity of the enzymes was assessed using SDS-PAGE with silver staining (Figs. S7 and S8). Enzyme concentration was determined by absorbance (280 nm) (39, 58) using extinction coefficients of ε^280^ = 23.4 mM^−1^·cm^−1^ for TDG^82-340^ (and E310Q-TDG^82-340^), ε^280^ = 27.9 mM^−1^·cm^−1^ for SUMO-1 modified TDG^82-340^, ε^280^ = 33.4 mM^−1^·cm^−1^ for TDG, and ε^280^ = 37.8 mM^−1^·cm^−1^ for SUMO-1 modified TDG. The purified enzyme was flash-frozen and stored at −80 °C.

### Oligodeoxynucleotides and duplex DNA

Standard (unmodified) oligodeoxynucleotides (ODNs) were obtained from IDT. ODNs containing 2′-fluoroarabino-dT, 2′-fluoroarabino-dU, 5-formylcytosine, or 5-carboxylcytosine were synthesized at the Keck Foundation Biotechnology Resource Laboratory of Yale University using phosphoramidites obtained from Glen Research. TDG binds productively to DNA containing 2′-fluoroarabino analogs, which are fully resistant to *N*-glycosyl bond cleavage because the fluorine substitution destabilizes the transition state of the glycosylase reaction (39, 43, 46, 49, 52, 59). ODNs were purified by reverse phase HPLC using an XBridge OST C18 column (Waters Corp) with mobile phases of 0.1 M TEAA pH 7.0 (A) and acetonitrile (B), a flow rate of 2.5 ml/min, and a gradient of 6 to 17% B over 30 min (60). ODN purity was assessed by anion-exchange HPLC under denaturing (pH 12) conditions using a DNAPac PA200 RS column (Thermo) (61). Purified ODNs were exchanged into 0.01 M Tris–HCl pH 8, 0.04 M NaCl, and 1 mM EDTA; the ODN concentration was determined by absorbance (A_260_) (41). The 28 bp duplex DNA contained a target strand, 5′-ACC AGT CCA TCG CTC A x G TAC AGA GCT G, where x = T, U, T^F^, U^F^, fC, caC, or C, and a complementary strand that pairs the target base (x) with G. We also obtained target strand ODNs that contained 3′-6-FAM (fluorescein) from IDT or we added a 3′-terminal fluorescein-dU using Fluorescein-12-ddUTP (Enzo) and terminal transferase (NEB). The 28 bp duplexes were prepared by mixing the target and complementary strands, heating to 80 °C, and slowly cooling to room temperature.

### Glycosylase activity assays

The single turnover reactions were collected at 37 °C by adding an enzyme to HEN.1 buffer (0.02 M HEPES pH 7.5, 0.1 M NaCl, 0.2 mM EDTA) that contained DNA substrate (0.2 nM–1.6 nM), BSA (0.1 mg/ml), and 28 bp nonspecific DNA with a G·C pair at the target site (250 nM). The enzyme concentration varied, with the lowest concentration at least 4-fold above the substrate concentration. Reaction samples were rapidly halted at specific time points by adding quench solution (0.1 M NaOH, 0.01 M EDTA, final). Quenched samples were heated for 3 to 5 min at 85 °C to quantitatively cleave the DNA backbone at the glycosylase-generated abasic site, and the resulting mixture of DNA fragments were resolved by anion-exchange HPLC under denaturing (pH 12) conditions using a DNAPac PA200 RS column (Thermo) (61). The integrals for peaks corresponding to intact and cleaved target strands were used to determine the fraction product for a given reaction sample (61). The progress curves (fraction product *vs.* time) were fitted to a single exponential equation (Equation 1) using non-linear regression:

(1) $$\text{fraction}\;\text{product}=A\left(1-\exp\left(-k_{\text{obs}}t\right)\right)$$

where *A* is the amplitude, *k*_obs_ is the rate constant, and *t* is reaction time. The hyperbolic dependence of *k*_obs_ on [E] was fitted to Equation 2 using non-linear regression:

(2) $$k_{\text{obs}}={\;k}_{\max}\;\left[E\right]/\left(\left[E\right]+K_{0.5}\right)$$

where *k*_max_ is the maximal rate of activity and *K*_0.5_ is the enzyme concentration [E] that gives half-maximal activity (*k*_obs_ = 0.5*k*_max_).

### ^19^F NMR spectroscopy

Fluorine NMR experiments were performed on a Bruker 600 MHz spectrometer (564.2 MHz for ^19^F) equipped with four channels, a Z-axis gradient, and a 5 mm HFCN cryogenic probe (optimized for ^1^H, ^19^F, ^13^C and ^15^N). The samples contained 58 to 75 μM DNA and a given TDG construct at a 2.7 to 3.0 M excess. TDG-free samples contained 45 to 50 μM DNA. All samples were in a buffer consisting of 15 mM Tris-HCl pH 7.5, 0.1 M NaCl, 0.03 mM TCEP, and 10% D_2_O. The ^19^F NMR experiments were collected with 8192 complex points, an acquisition time of 0.66 s, and a relaxation delay of 2.0 s. The experiments were collected with 5000 scans for free DNA and 16,000 scans for protein-DNA complexes. The data were processed by applying exponential multiplication with 25 Hz line broadening prior to Fourier transformation and baseline correction using TopSpin (Bruker). The observed ^19^F chemical shifts (*δ*^19^F) are relative to an external sample of trifluoroacetic acid (6.5 mM) in the same buffer.

## Data availability

The data supporting the findings of this study are available within the main article or the supporting information.

## Supporting information

This article contains supporting information.

## Conflict of interest

The authors declare that they have no conflicts of interest with the contents of this article.
