# Supplementary material for: Sumoylation of thymine DNA glycosylase impairs productive binding to substrate sites in DNA
Source: J Biol Chem. 2024 Oct 18;300(11):107902. doi: 10.1016/j.jbc.2024.107902 (PMC11602971; doi:10.1016/j.jbc.2024.107902)
Supplement: Supporting Information [file mmc1.pdf]

Supporting Information

Sumoylation of thymine DNA glycosylase impairs productive binding to substrate sites in DNA

Lakshmi S. Pidugu,[1] Hardler W. Servius,[1] Kurt B. Espinosa,[1] Mary E. Cook,[1] Kristen M. Varney,[1] and Alexander C. Drohat[1,2,*]

[1]Department of Biochemistry and Molecular Biology, University of Maryland School of Medicine, Baltimore, MD 21201, USA, [2]Molecular and Structural Biology Program, University of Maryland Marlene and Stewart Greenebaum Comprehensive Cancer Center, Baltimore, MD 21201, USA

* To whom correspondence should be addressed. Tel: 410-706-8118; Email: adrohat@som.umaryland.edu

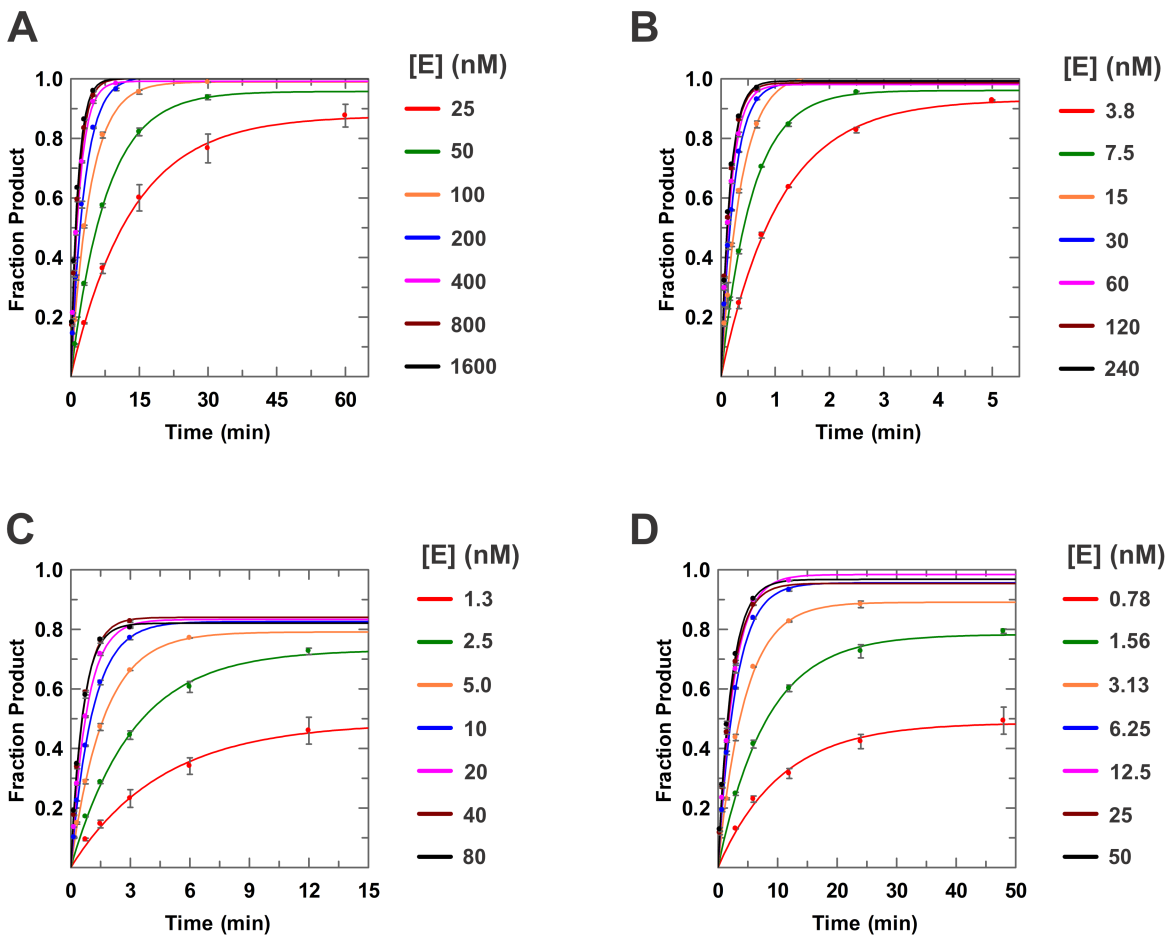

Figure S1. Glycosylase activity for TDG acting on DNA substrates including (A) G·T, (B) G·U, (C) G·fC, and (D) G·caC. Single turnover kinetics experiments were performed (at 37 °C) using a limiting substrate concentration (0.2 nM) and the indicated enzyme concentration. Provided below are k_{obs} values for each enzyme concentration (in parentheses).

(A) G·T: k_{obs} = 0.076 ± 0.006 (25 nM), 0.130 ± 0.002 (50 nM), 0.236 ± 0.005 (100 nM), 0.336 ± 0.009 (200 nM), 0.520 ± 0.008 (400 nM), 0.586 ± 0.013 (800 nM), 0.654 ± 0.014 (1600 nM)

(B) G·U: k_{obs} = 0.927 ± 0.022 (3.8 nM), 1.76 ± 0.04 (7.5 nM), 2.72 ± 0.26 (15 nM), 4.27 ± 0.09 (30 nM), 5.47 ± 0.09 (60 nM), 6.07 ± 0.10 (120 nM), 6.14 ± 0.11 (240 nM)

(C) G·fC: k_{obs} = 0.219 ± 0.035 (1.3 nM), 0.318 ± 0.015 (2.5 nM), 0.606 ± 0.010 (5.0 nM), 0.916 ± 0.026 (10 nM), 1.24 ± 0.04 (20 nM), 1.55 ± 0.04 (40 nM), 1.65 ± 0.04 (80 nM)

(D) G·caC: k_{obs} = 0.095 ± 0.010 (0.78 nM), 0.123 ± 0.005 (1.6 nM), 0.223 ± 0.007 (3.1 nM), 0.334 ± 0.008 (6.3 nM), 0.379 ± 0.008 (12.5 nM), 0.429 ± 0.008 (25 nM), 0.450 ± 0.012 (50 nM)

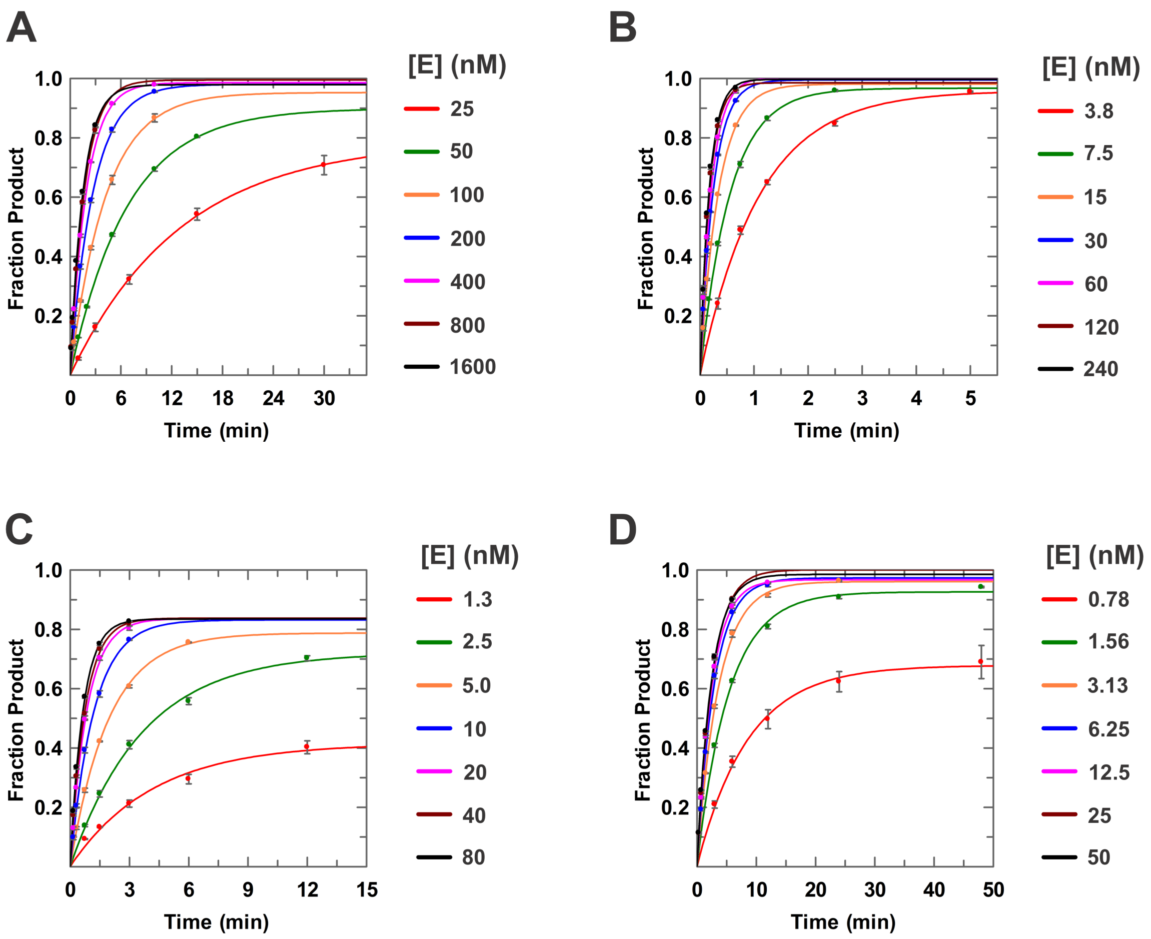

Figure S2. Glycosylase activity for TDG$^{82-340}$ acting on DNA substrates including (A) G·T, (B) G·U, (C) G·fC, and (D) G·caC. Single turnover kinetics experiments were performed (at 37 °C) using a limiting substrate concentration (0.2 nM) and the indicated enzyme concentration. Provided below are k_{obs} values for each enzyme concentration (parentheses).

(A) G·T: k_{obs} = 0.076 ± 0.006 (25 nM), 0.148 ± 0.002 (50 nM), 0.238 ± 0.007 (100 nM), 0.369 ± 0.005 (200 nM), 0.518 ± 0.004 (400 nM), 0.588 ± 0.014 (800 nM), 0.658 ± 0.016 (1600 nM)

(B) G·U: k_{obs} = 0.909 ± 0.026 (3.8 nM), 1.80 ± 0.03 (7.5 nM), 2.92 ± 0.09 (15 nM), 4.03 ± 0.08 (30 nM), 4.73 ± 0.09 (60 nM), 5.69 ± 0.16 (120 nM), 5.8214 ± 0.20 (240 nM)

(C) G·fC: k_{obs} = 0.239 ± 0.028 (1.3 nM), 0.271 ± 0.013 (2.5 nM), 0.512 ± 0.012 (5 nM), 0.826 ± 0.028 (10 nM), 1.17 ± 0.04 (20 nM), 1.33 ± 0.03 (40 nM), 1.54 ± 0.02 (80 nM)

(D) G·caC: k_{obs} = 0.116 ± 0.011 (0.78 nM), 0.186 ± 0.005 (1.6 nM), 0.274 ± 0.005 (3.1 nM), 0.343 ± 0.012 (6.3 nM), 0.394 ± 0.007 (12.5 nM), 0.394 ± 0.009 (25 nM), 0.412 ± 0.010 (50 nM)

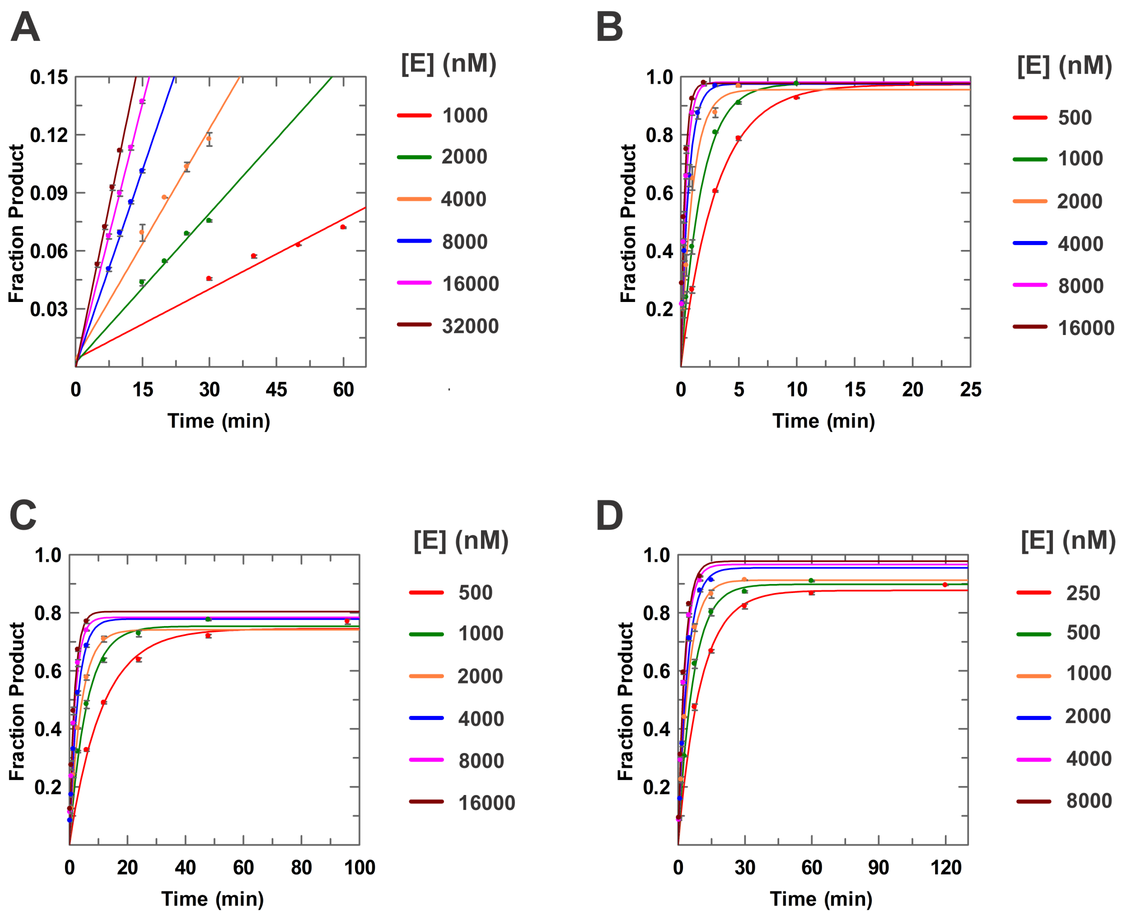

Figure S3. Glycosylase activity for sumoylated TDG on DNA substrates including (A) G·T, (B) G·U, (C) G·fC, and (D) G·caC. Single turnover kinetics experiments were performed (at 37 °C) using a limiting substrate concentration (1.6 nM) and the indicated enzyme concentration. Because the activity for the G·T substrate was too slow for fitting to a single exponential equation (eq. 1), the sample time points were selected to give no more than ~15% product and the progress curves were fitted to a linear equation, where k_{obs} is given by the slope. Provided below are k_{obs} values for each enzyme concentration (parentheses).

(A) G·T: k_{obs} = 0.0012 ± 0.0001 (1 μM), 0.0026 ± 0.0001 (2 μM), 0.0040 ± 0.0002 (4 μM), 0.0068 ± 0.0001 (8 μM), 0.0091 ± 0.0001 (16 μM), 0.0112 ± 0.0002 (32 μM)

(B) G·U: k_{obs} = 0.324 ± 0.005 (0.5 μM), 0.561 ± 0.018 (1 μM), 1.01 ± 0.08 (2 μM), 1.53 ± 0.08 (4 μM), 2.23 ± 0.04 (8 μM), 2.96 ± 0.11 (16 μM)

(C) G·fC: k_{obs} = 0.089 ± 0.004 (0.5 μM), 0.171 ± 0.007 (1 μM), 0.256 ± 0.006 (2 μM), 0.361 ± 0.013 (4 μM), 0.504 ± 0.017 (8 μM), 0.568 ± 0.023 (16 μM)

(D) G·caC: k_{obs} = 0.099 ± 0.003 (0.25 μM), 0.149 ± 0.005 (0.5 μM), 0.215 ± 0.008 (1 μM), 0.242 ± 0.017 (2 μM), 0.318 ± 0.024 (4 μM), 0.343 ± 0.028 (8 μM)

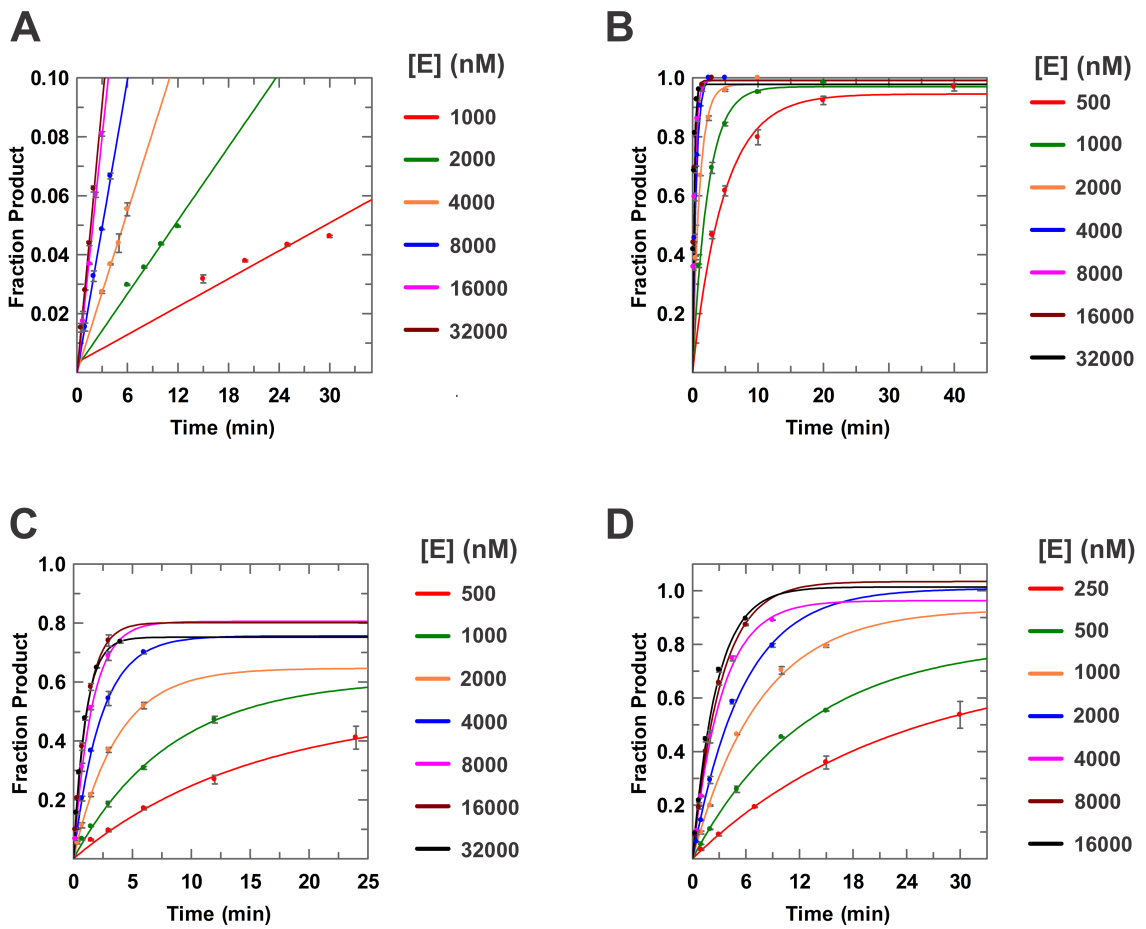

Figure S4. Glycosylase activity for sumoylated TDG$^{82\text{-}340}$ acting on DNA substrates including (A) G·T, (B) G·U, (C) G·fC, and (D) G·caC. Single turnover kinetics experiments were performed (at 37 °C) using a limiting substrate concentration (1.6 nM) and the indicated enzyme concentration. Because the activity for the G·T substrate was too slow for fitting to a single exponential equation (eq. 1), the sample time points were selected to give no more than ~15% product and the progress curves were fitted to a linear equation, where k_{obs} is given by the slope. Provided below are k_{obs} values for each enzyme concentration (parentheses).

(A) G·T: k_{obs} = 0.0016 ± 0.0001 (1 μM), 0.0042 ± 0.0002 (2 μM), 0.0091 ± 0.0003 (4 μM), 0.0166 ± 0.0003 (8 μM), 0.0273 ± 0.0007 (16 μM), 0.0306 ± 0.0008 (32 μM)

(B) G·U: k_{obs} = 0.210 ± 0.010 (0.5 μM), 0.429 ± 0.014 (1 μM), 0.935 ± 0.031 (2 μM), 1.79 ± 0.03 (4 μM), 2.72 ± 0.05 (8 μM), 3.57 ± 0.05 (16 μM), 4.39 ± 0.10 (32 μM)

(C) G·fC: k_{obs} = 0.066 ± 0.011 (0.5 μM), 0.122 ± 0.010 (1 μM), 0.274 ± 0.016 (2 μM), 0.431 ± 0.018 (4 μM), 0.652 ± 0.036 (8 μM), 0.862 ± 0.035 (16 μM), 0.987 ± 0.016 (32 μM)

(D) G·caC: k_{obs} = 0.045 ± 0.008 (0.25 μM), 0.079 ± 0.007 (0.5 μM), 0.133 ± 0.009 (1 μM), 0.178 ± 0.016 (2 μM), 0.309 ± 0.020 (4 μM), 0.318 ± 0.019 (8 μM), 0.373 ± 0.023 (16 μM)

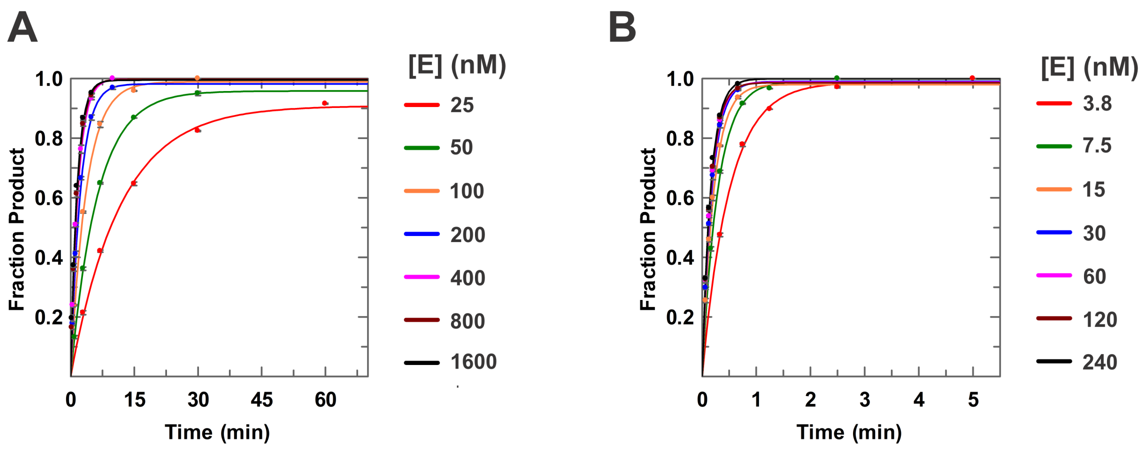

Figure S5. Glycosylase activity for E310Q-TDG$^{82\text{-}340}$ acting on DNA substrates including (A) G·T, and (B) G·U. Single turnover kinetics experiments were performed (at 37 °C) using a limiting substrate concentration (0.2 nM) and the indicated enzyme concentration. Provided below are k_{obs} values for each enzyme concentration (parentheses).

(A) G·T: k_{obs} = 0.085 ± 0.002 (25 nM), 0.159 ± 0.002 (50 nM), 0.273 ± 0.005 (100 nM), 0.438 ± 0.010 (200 nM), 0.565 ± 0.010 (400 nM), 0.614 ± 0.020 (800 nM), 0.664 ± 0.018 (1600 nM)

(B) G·U: k_{obs} = 1.99 ± 0.04 (3.8 nM), 3.47 ± 0.06 (7.5 nM), 4.69 ± 0.08 (15 nM), 5.56 ± 0.12 (30 nM), 5.97 ± 0.07 (60 nM), 6.22 ± 0.10 (120 nM), 6.30 ± 0.13 (240 nM)

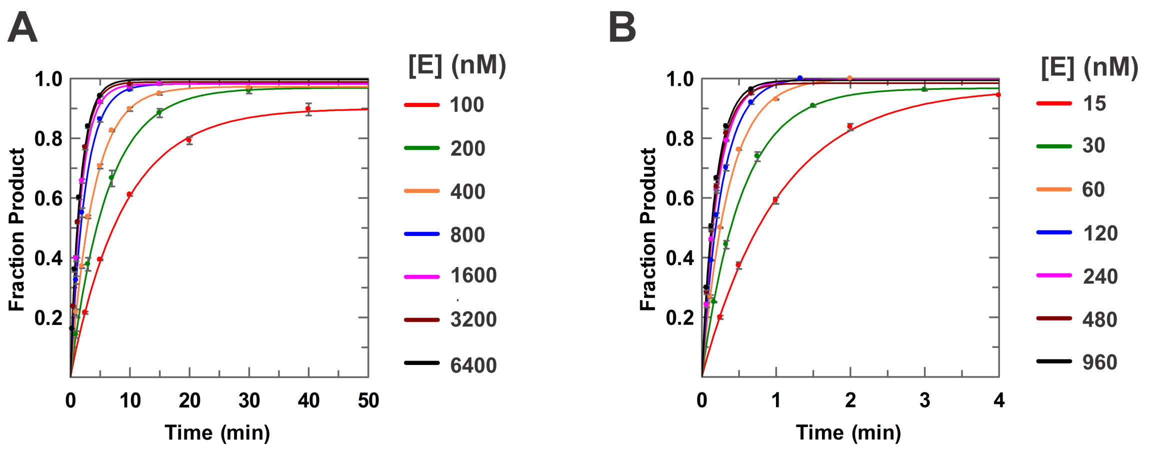

Figure S6. Glycosylase activity for sumoylated E310Q-TDG$^{82\text{-}340}$ acting on DNA substrates including (A) G·T and (B) G·U. Single turnover kinetics experiments were performed (at 37 °C) using a limiting substrate concentration (0.2 nM) and the indicated enzyme concentration. Provided below are k_{obs} values for each enzyme concentration (parentheses).

(A) G·T: k_{obs} = 0.112 ± 0.003 (100 nM), k_{obs} = 0.170 ± 0.005 (200 nM), k_{obs} = 0.247 ± 0.005 (400 nM), 0.410 ± 0.008 (800 nM), 0.540 ± 0.009 (1600 nM), 0.590 ± 0.010 (3200 nM), 0.603 ± 0.017 (6400 nM)

(B) G·U: k_{obs} = 0.958 ± 0.023 (15 nM), 1.86 ± 0.04 (30 nM), 2.77 ± 0.04 (60 nM), 3.71 ± 0.07 (120 nM), 4.65 ± 0.20 (240 nM), 5.20 ± 0.08 (480 nM), 5.43 ± 0.11 (960 nM)

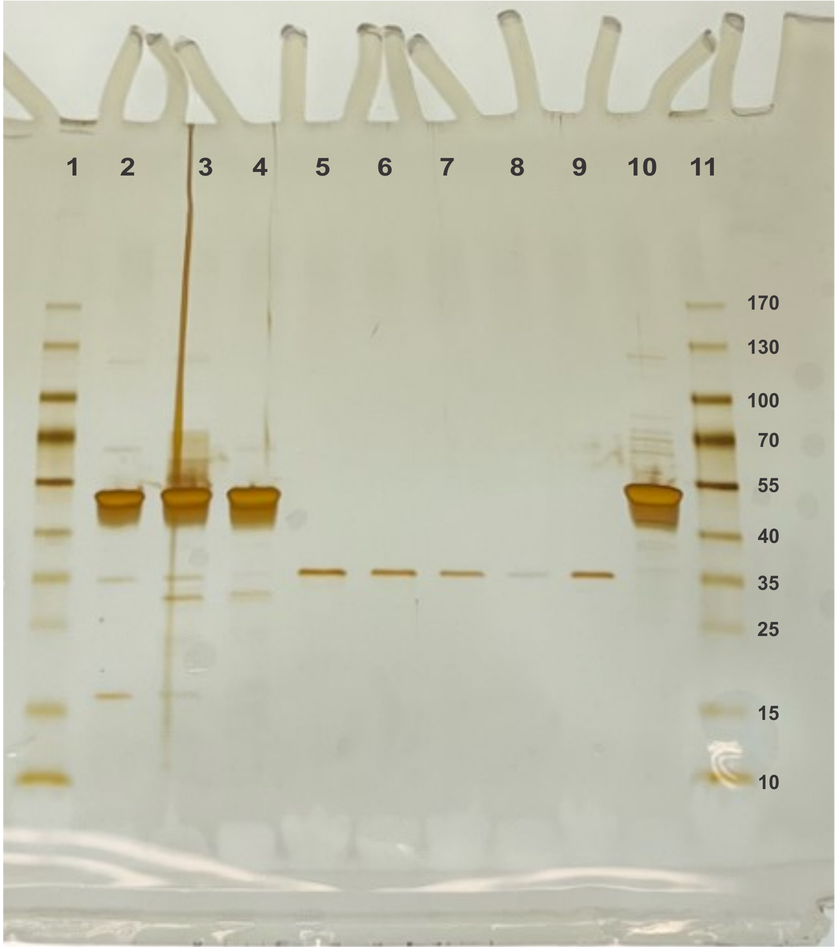

Figure S7. SDS-PAGE analysis of sumoylated TDG$^{82\text{-}340}$, sumoylated E310Q-TDG$^{82\text{-}340}$, and unmodified TDG$^{82\text{-}340}$. Lanes 2-4 contain 20 picomol (2 µl, 10 µM) of sumoylated E310Q-TDG$^{82\text{-}340}$ used for NMR with G·T^{F} DNA (lane 2), NMR with G·U^{F} DNA (lane 3), and the glycosylase activity assays (lane 4). Lanes 5-9 contain unmodified TDG$^{82\text{-}340}$ in the amounts of 0.4 picomol (lane 5), 0.3 picomol (lane 6), 0.2 picomol (lane 7), 0.1 picomol (lane 8), and 0.4 picomol (lane 9). Lane 10 contains 20 picomol of sumoylated TDG$^{82\text{-}340}$ used for NMR samples. Lanes 1 and 11 contain PageRuler Protein Ladder (Thermo); 2 µl diluted 50-fold. The gel was stained by silver staining (Pierce). The results indicate that the samples of sumoylated TDG$^{82\text{-}340}$ and sumoylated E310Q-TDG$^{82\text{-}340}$ contain no more than 1% of the unmodified form of TDG$^{82\text{-}340}$ or E310Q-TDG$^{82\text{-}340}$.

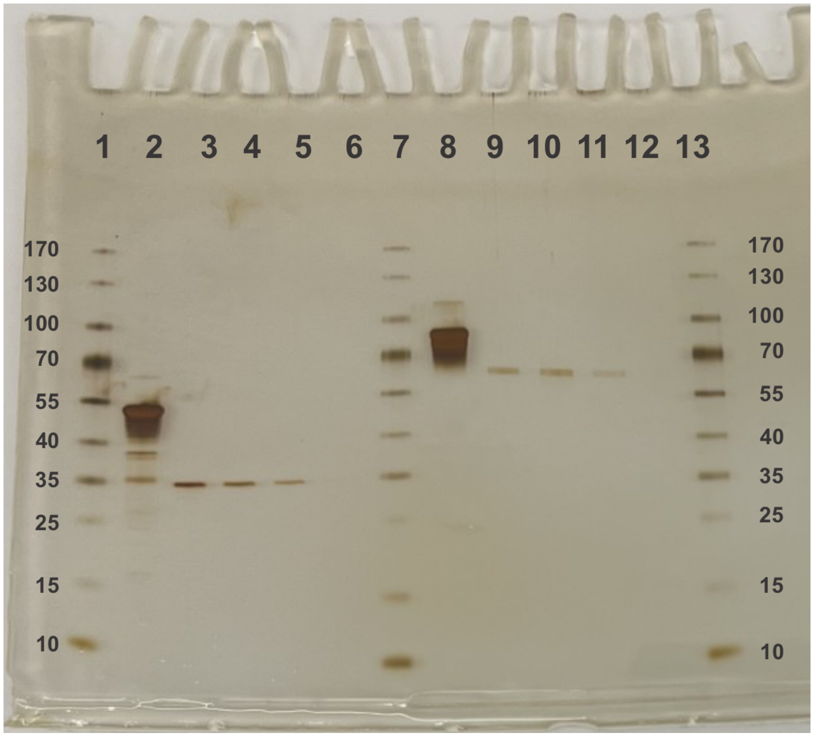

Figure S8. SDS-PAGE analysis of purified sumoylated and unmodified forms of TDG and TDG$^{82\text{-}340}$. Lane 2 contains 20 picomol (2 µl, 10 µM) of sumoylated TDG$^{82\text{-}340}$. Lanes 3-6 contain unmodified TDG$^{82\text{-}340}$ in the amounts of 0.4 picomol (lane 3), 0.3 picomol (lane 4), 0.2 picomol (lane 5), and 0.1 picomol (lane 6). Lane 8 contains 20 picomol (2 µl, 10 µM) of sumoylated TDG. Lanes 9-12 contain unmodified TDG in the amounts of 0.4 picomol (lane 9), 0.3 picomol (lane 10), 0.2 picomol (lane 11), and 0.1 picomol (lane 12). Lanes 1, 7, and 12 contain PageRuler Protein Ladder (Thermo; 2 µl diluted 50-fold). The gel was stained by silver staining (Pierce). The results indicate that the samples of sumoylated TDG$^{82\text{-}340}$ and sumoylated TDG contain less than 1% of unmodified TDG$^{82\text{-}340}$ and TDG, respectively. We note that the minor contaminant in the sumoylated TDG$^{82\text{-}308}$ sample (lane 2) migrates slightly slower than the 35 kDa while unmodified TDG$^{82\text{-}308}$ migrates slightly faster than that marker.
